# Radiography Finding in the Jaws in Children Taking Bisphosphonate

**Published:** 2013-07-22

**Authors:** M Moeini, M Moeini, N Lotfizadeh, M Alavi

**Affiliations:** 1 Resident of Oral and Maxillofacial Radiology, Faculty of Dentistry, Shahid Sadoughi University of Medical Science and Health Services, Yazd, Iran.; 2 Dentist, Esfahan, Iran.; 3 Dentist, Tehran, Iran.; 4 Student, Faculty of Dentistry, Shahid Sadoughi University of Medical Science and Health Services, Yazd, Iran.‎

**Keywords:** Osteonecrosis, Jaw, Radiography, Thalassemia

## Abstract

**Background:**

Bisphosphonates‎ inhibit osteoclasts, prevent bone resorption and decrease bone turnover. This study examined radiography finding in bisphosphonate-associated osteonecrosis of jaws.

**Materials and Methods:**

This is a retrospective series of 12 clinically diagnosed patients between 7 to 21 year old (average 13 years). They required emergency dental conditions requiring management by dentist: non healing ‎extraction sockets and pain of bone exposure. The panoramic radiography and cone beam computed tomography was performed ‎to assess the problem.

Thickening of the lamina dura was observed in 7 patients (58.3 %). But full-thickness sclerosis was seen in 6 patients (50 %). Sclerotic changes in the mandibular canal were noted in 3 patients (25 %). 5 patients (41.6 %) had poorly healing or non-healing of socket in extracted tooth and periapical lucencies.

4 people (33.3 %) had widening of periodontal ligament (PDL) space and osteolysis. Sequestra were seen in 3 persons (25 %). Finally in 2 children (16.6 %) were found oroantral fistula. Only one child (8.3 %) had thickening of soft tissue mid periosteal reaction.

**Results:**

Most patients had some degree of osteosclerosis, especially in the area of alveolar bone. Thickening of the lamina dura was also seen in children. Other findings include: osteolysis, sequestra, periosteal new bone formation, widening of PDL,soft tissue thickening, non healing extraction sockets, oroantral fistula and periapical lucencies (P-value < 0.05).

**Conclusion:**

Common radiographic features in patients taking bisphosphonate, was osteosclerosis. This sclerosis had different views that thickening of the lamina dura and alveolar crest were most common.

## Introduction

Bisphosphonates are drugs that are used for the following diseases such as: osteoporosis, Paget's disease, osseous metastases, osteogenesis imperfecta, thalassemia major, and multiple myeloma, rheumatoid arthritisand malignancy-related hypercalcemia (1, 2, 3, and 4). The bisphosphonates inhibit osteoclasts, inhibit bone resorption and decrease bone turnover (‎^5^, ^6^, ^7^, ^8^, ^9^‎). In 2002, the relationship between osteonecrosis of the jaw and bisphosphonates was proved (10, 11, and 12). These patients often complain about painful bone exposure and non healing extraction socket (13, 14, 15, 16‎). Nowadays due to the increasing incidence of malignancies and increasing the use of bisphosphonates, side effects of these drugs should be investigated further. Bisphosphonate-associated osteonecrosis of jaws (BOJ)is diagnosed through: clinical examination of the patient's mouth and radiography because these patients have problem in healing postsurgical intervention and biopsy site (17, 18). So radiography and radiographic findings has great role in BOJ diagnosis. In this study we investigated all of radiographic findings in BOJ. 

Common radiographic features are "osteosclerosis" (sclerosing of the alveolar margin and thickening of the lamina dura). In more sever patients higher degrees of sclerosis were seen, which were very similar to osteopetrosis. "Narrowing of the inferior alveolar canal" was seen in large lesions. Other radiographic findings with lower incidence are: osteolysis, periosteal reaction, oroantral fistula, sequestra, periapical lucency and swelling soft tissue. To our knowledge, few papers have been published about radiographic findings of BOJ. 

## Materials and Methods

In this study we assess 12 children taking bisphosphonates (in Esfahan city, Iran) who had osteonecrosis of the jaws (from April 2012 until May 2013) and were diagnosed clinically. These patients received bisphosphonate therapyand meanwhile they required emergency dental conditions: non healing extraction sockets, pain of bone exposure, infection difficult to manage with conventional treatment which was requiring management by dentist. So the panoramic radiography and cone beam computed tomography (CBCT) was taken to findingthe reasons. Two radiologists reviewed the imaging of the jaws.

## Results

Nine patients were women. All of patients had prostate thalassemia major. The age range of the patients (at the first time seen by a dentist) was between 7 to 21 year old (average, 13 years). The mandible was the clinical site of involvement in 9 patients (75 %), and the maxilla was involved in 3 patients (25 %). All patients in our study had some degree of BOJ. Most of them were involved in alveolar margin. Thickening of the lamina dura was observed in 7 patients (58.3 %). But full-thickness sclerosis was seen in 6 patients (50 %).Sclerotic changes in the mandibular canal were noted in 3 patients (25 %). 5 patients (41.6 %) had poorly healing or non-healing of socket in extracted tooth and periapical lucencies. 4 people (33.3 %) had widening of periodontal ligament (PDL) space and osteolysis. Sequestra were seen in 3 persons (25 %). Finally in 2 children (16.6 %) were found oroantral fistula. Only one child (8.3 %) had thickening of soft tissue associated with periosteal reaction.

**Radiographic Findings**

**Figure 1:**
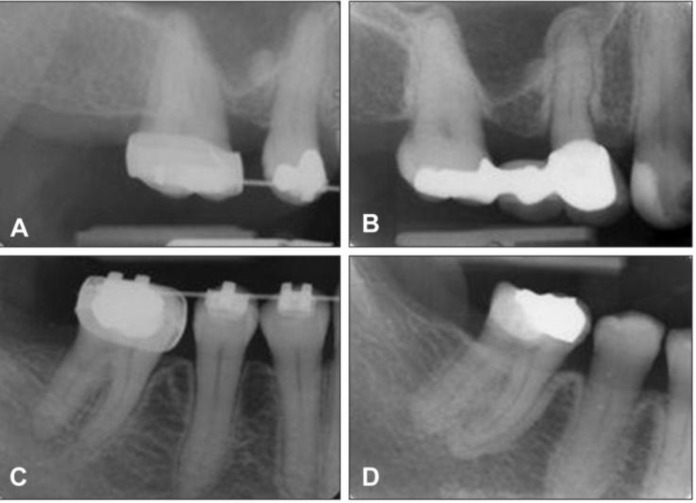
Periapical radiographs of a woman, showing generalized osseous sclerosis ‎of uniform thickness involving the cortical plate and lamina dura. A and C radiographs shows the ‎pre-bisphosphonate therapy condition of the cortical plates and the lamina dura. B and D ‎radiographs shows thickening of the cortical plate and lamina dura after 2 years bisphosphonate ‎therapy. No clinical signs and symptoms were established at this initial stage

**Figure 2:**
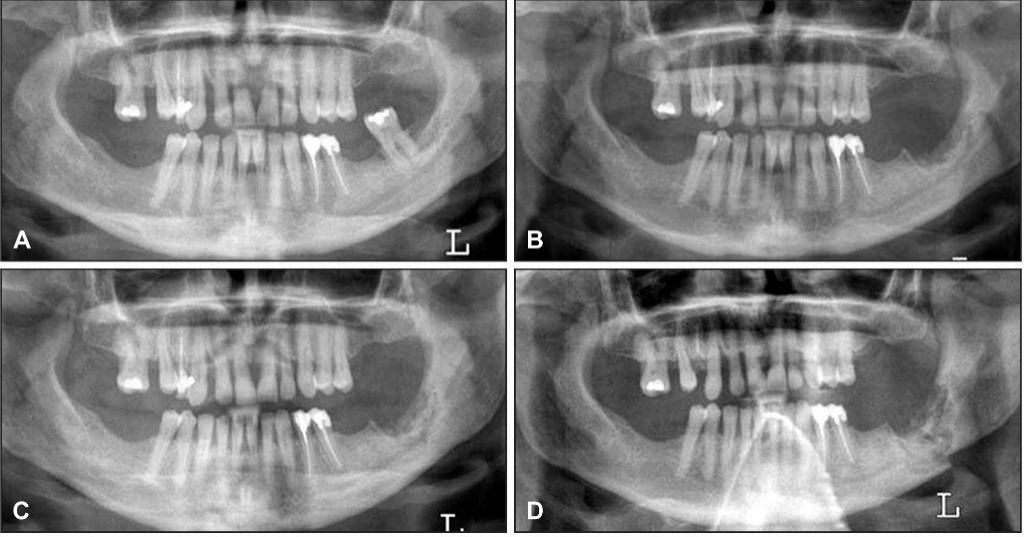
A = Panoramic radiograph of a woman with breast cancer treated with zoledronate ‎who presented with a painful tooth # 37‎‏.‏ B = Panoramic radiograph after 7 months, showing non healing extraction site in the left ‎posterior mandible, absence of bone remodeling and sclerotic bone changes of the body of the ‎mandible‏.‏ C = Panoramic radiograph after 9 months demonstrates a nonhealing extraction site in the left ‎posterior mandible with progressive sclerosis of the left body and angle of the mandible with ‎encroachment on the left mandibular canal‏.‏ D = Panoramic radiograph after 19 months with intervening curettage, demonstrates progression ‎of sclerosis to pathologic fracture of the mandible.

**Figure 3:**
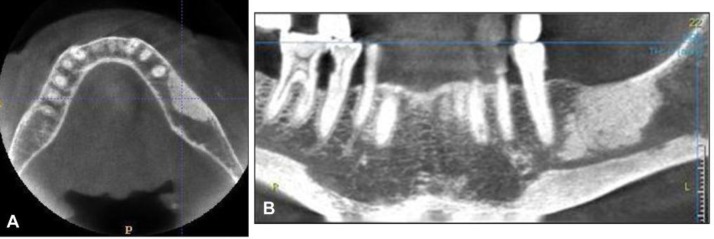
A = Axial images of the mandible obtained with CBCT unit showing thickening of ‎cortical plate and focal region of medullary bone density on the left body of mandible, the ‎overlying mucosa is clinically intact‏.‏ B = Two-dimensional multiplanar reconstruction image showing the vertical dimension of the ‎region of osteosclerosis and the relationship with mandibular canal.

Common radiographic features were "osteosclerosis" (sclerosing of the alveolar margin and thickening of the lamina dura) similar to the study by Marx et al (19) (figure 1). Usually sizes of bone defects are larger than clinical lesions. In more advanced patients were seen higher degrees of sclerosis (figure 2, 3), which were very similar to osteopetrosis. In large lesions was seen "narrowing of the inferior alveolar canal". We think that generalized nature of BOJ help radiologist to recognize these lesions from localized reactive sclerosis around the inflammatory center. 

Other radiographic findings with lower incidence are: "osteolysis", "periosteal reaction", "oroantral fistula", "sequestra", "periapical lucency" and "swelling soft tissue". To our knowledge, few papers have been presented about radiographic findings of BOJ. Most authors described lytic degenerative changes, sequestra and swelling soft tissue‎. Until Marx et al. mentioned sclerotic change in jaws (19).

Differential diagnoses for BOJ are osteoradionecrosis, chronic sclerotic osteomyelitis, metastatic carcinoma, Paget's disease and osteopetrosis. In osteoradionecrosis, patient always has a history of radiotherapy and the borders of these lesions in radiographic feature have poorly defines.Chronic sclerotic osteomyelitis has several radiologic hallmarks: sequestra, periosteal reaction, sclerosis and bone expansion. Metastases often occur in the posterior mandible that formsmultiple lytic lesions except prostate and breast metastatic cancers which can be sclerotic. On the otherPaget's disease has bone expansion and coarse trabecular. 

We can define superimposed infection on BOJ by radiographic view. Also it can be used for staging of BOJ (17). 

CT scan especially CBCT has highest sensitivity than panoramic radiography the swelling soft tissue, periosteal reaction and sequestra are easierdiagnosed by CT (17, 20). 

Finally, the main limitation of our study was low number of patients. We suggest that in future studies have larger samples.

## Discussion

In normal bone, there is a balance between bone formation and resorption. Bisphosphonates are drugs that reduce bone resorption by inhibiting osteoclasts. BOJ is a known health crisis that is due to bisphosphonate use.

Specific clinical evidence to determine is bone exposure with or without pain and history of bisphosphonate‎s usage. In these patients biopsy should be avoided. Since these patients have a fundamental problem in bone repair, biopsy is recommended only when they are suspected to metastasis.

It should be noted thatBOJ can occur long time after the treatment with bisphosphonates. Bisphosphonates skeletal half-life is 12 years.T hemechanism of BOJ is very similar to osteopetrosis since it has a defect in bone resorption too.Clinical effects of osteopetrosis in jaws are like to BOJ such as problem in healing and infection of jaws after dental procedures. 

Betweenall bones of the body,jaws bones (maxilla and mandible) ‎are more sensitive to the effects of bisphosphonates because they have high bone turnover. A few examples of the BOJare reported out of the oral cavity.

BOJ diagnosis is based on clinical principles: clinical examination, radiography and history of bisphosphonate use. 

## Conclusion

Common radiographic features in patients (children) taking bisphosphonate, was osteosclerosis. This sclerosis had different views than thickening of the lamina dura and alveolar crest in jaws were most common. 
